# Category variability effect in category learning with auditory stimuli

**DOI:** 10.3389/fpsyg.2014.01122

**Published:** 2014-10-02

**Authors:** Lee-Xieng Yang, Yueh-Hsun Wu

**Affiliations:** ^1^Department of Psychology and Research Center for Mind, Brain, and Learning, National Chengchi UniversityTaipei, Taiwan; ^2^Department of Psychology, National Chengchi UniversityTaipei, Taiwan

**Keywords:** category variability effect, sequence effect, perceptual category learning, memory and comparison, decision bound model

## Abstract

The category variability effect refers to that people tend to classify the midpoint item between two categories as the category more variable. This effect is regarded as evidence against the exemplar model, such as GCM (Generalized Context Model) and favoring the rule model, such as GRT (i.e., the decision bound model). Although this effect has been found in conceptual category learning, it is not often observed in perceptual category learning. To figure out why the category variability effect is seldom reported in the past studies, we propose two hypotheses. First, due to sequence effect, the midpoint item would be classified as different categories, when following different items. When we combine these inconsistent responses for the midpoint item, no category variability effect occurs. Second, instead of the combination of sequence effect in different categorization conditions, the combination of different categorization strategies conceals the category variability effect. One experiment is conducted with single tones of different frequencies as stimuli. The collected data reveal sequence effect. However, the modeling results with the MAC model and the decision bound model support that the existence of individual differences is the reason for why no category variability effect occurs. Three groups are identified by their categorization strategy. Group 1 is rule user, placing the category boundary close to the low-variability category, hence inducing category variability effect. Group 2 takes the MAC strategy and classifies the midpoint item as different categories, depending on its preceding item. Group 3 classifies the midpoint item as the low-variability category, which is consistent with the prediction of the decision bound model as well as GCM. Nonetheless, our conclusion is that category variability effect can be found in perceptual category learning, but might be concealed by the averaged data.

The seminal study of Rips (1989) showed that people tend to classify an item (e.g., a 3-inches circular object) at the midpoint between two categories (e.g., QUATER and PIZZA) as the category with a larger variability (i.e., PIZZA), although the middle item is more similar to the low-variability category (i.e., QUATER). This finding attracts many researchers' attention, for it indicates that category variability is one of the sources for categorization and challenges the exemplar-based model, specifically GCM (Generalized Context Model; Nosofsky, 1986, 1987). Since the exemplars of low-variability category vary in a smaller range than those of high-variability category, the total distance from exemplars to the middle item is shorter for the low-variability category than the high-variability category. Thus, the middle item is more similar to the low-variability category. Based on similarity, GCM would always classify the middle item as the low-variability category. Only when the two categories in the same psychological space have different specificities for similarity computation, can GCM predict Rips (1989)' finding (see Nosofsky and Johansen, 2000).

In contrast, the famous rule-based model GRT (Generalized Recognition Theory; Ashby and Townsend, 1986; Ashby and Gott, 1988; Ashby and Maddox, 1992; Maddox and Ashby, 1993) is thought to be able to account for this phenomenon. According to GRT, learning categories is to generate a category boundary. The boundary divides the psychological space into different regions, each of which corresponds to a category. An item would be classified as a category, if its percept is located in the region corresponding to that category. Each category is assumed to be represented as a normal distribution with the mean location having the largest likelihood to be classified as that category. The optimal boundary between two categories is located on where the percept of item has an equally high likelihood to be classified as either category. According to the nature of normal distribution, the likelihood of a value is a function of the distribution variance. Thus, the optimal category boundary will be influenced by the variance of category distribution and always close to the low-variability category. This is why the middle item would be predicted as the high-variability category by GRT.

Although this phenomenon is observed in conceptual category learning, it is not often reported in the studies of perceptual category learning. Thus, the purpose of this study is to examine whether the variability of category would influence perceptual categorization. Specifically, how the midpoint item between two categories would be classified is our focus. For the convenience of discussion, we follow Stewart and Chater (2002) to call this phenomenon category variability effect (CVE). In the later sections, we review the past studies, discussing the possible reasons for the low reliability of them, including the sequence effect in category learning and individual differences, and then introduce our experiment, discussing the empirical data, and modeling results.

## 1. Category variability effect in perceptual category learning

In the study of Cohen et al. (2001), two categories were defined as high-variability and low-variability categories by their covering range on the stimulus dimension. In the learning phase, the participants learned to correctly classify the exemplars of these two categories. In the transfer phase, the critical item was presented for the participants to predict its category label. The results showed that the probability of high-variability category for the critical item became higher when the exemplar number of high-variability category increased from two to seven, with the exemplar number of low-variability category fixed to one. However, the probability of high-variability category for the critical item is still not significantly larger than 0.50, namely no CVE occurred.

Stewart and Chater (2002) used a circle with a dot attaching on its periphery as stimulus. The dot position was the stimulus dimension and the high-variability and low-variability categories, respectively, cover a larger and a smaller portion of the periphery. Their results showed no CVE when the participants were presented with one stimulus on each trial. However, when all exemplars of each category were presented together to the participants in the learning phase, CVE was observed. Thus, it seems critical to CVE that people should be aware of the variability of category.

Similar to Cohen et al. (2001), Hsu and Griffiths (2010) also used lines of different lengths as stimuli to examine CVE. In the discrimination condition, the participants were instructed to predict the category label, given the current line length. In the generation condition, the participants were instructed to predict which category would be more likely to have a line of this length. The results showed no CVE in the discrimination condition and a clear CVE in the generation condition. These behavioral results were correctly simulated by their Bayes network models. For the generative condition, the model aimed to estimate the probability distribution over the input given the category label, namely *p(x|c)*. However, for the discrimination condition, the model aimed to find a direct mapping between inputs and category labels, namely *p(c|x)*. The success of their models implied that the occurrence of CVE demands the knowledge about candidate categories. Together with the findings of Stewart and Chater (2002), this knowledge should include the variability of each category.

According to the previous review, it is not clear whether CVE would occur in category learning. To figure out why the past studies did not observe CVE is the purpose of this study. We seek for the answer by checking out the nature of category learning task, instead of testing people in some new experimental design. Our focus is on the sequence effect and individual differences in category learning.

## 2. Sequence effect in category learning

Normally, the category representation (i.e., rule or exemplars) is assumed to be quite stable during category learning, as it is the representation of category structure, which would not change throughout the experiment. Thus, with the stable category representation, one item would be classified to the same category under any circumstances. However, recent studies show that the same item might be classified as different categories when following different items (Stewart et al., 2002; Stewart and Brown, 2004). This finding instead suggests the possibility of short-term representation (i.e., the information of the preceding item) to be adopted in category learning. Inspired by this finding, in the case of CVE, the midpoint item may be classified as one category when following a certain items and the other category when following some other items. Accordingly, when mixing up these conditions, the averaged result would show no CVE. If this is true, we should expect some sequence effect in the experiment for examining CVE.

The sequence effect of our interest is suggested by Stewart et al. (2002)'s MAC (Memory and Comparison) strategy for categorization. The MAC strategy is very simple. Suppose we know that one category takes larger values and the other takes smaller values, just like the one-dimensional category structure used for examining CVE. When item *n* − 1 is from the large category and item *n* is even larger than it, *X_n_* ≥ *X*_*n*−1_, item *n* must be the large category. Likewise, when item *n* − 1 item is from the small category and *X_n_* ≤ *X*_*n*−1_, item *n* must also be the small category. That is, when the sign of the difference between successive items can guarantee the category of the latter one, the probability to repeat the preceding category label as the response for the latter item is 1.00. When this heuristic cannot be applies to categorization, that is *X_n_* < *X*_*n*−1_ when item *n* − 1 is from the large category or *X_n_* > *X*_*n*−1_ when item *n* − 1 is from the small category, the probability to repeat the preceding category as the current response is the similarity between item *n* − 1 and item *n*. The MAC model can be expressed as

(1) $$p=\{{}_{exp}\overset{1.00}{{}^{-c|X_{n}-X_{n-1}|}},$$

where *c* is the specificity, when *c* is large, items would be less similar and vice versa. The similarity between item *n* − 1 and item *n* is exponentially transferred from their psychological distance. The smaller the distance, the larger the similarity.

According to the MAC strategy, we define the sequence effect as the tendency to repeat the preceding category label as current response. In this study, we would like to examine the sequence effect in categorization. Specifically, we would like to check if this effect is the reason for the inconsistent reports about CVE in the past studies.

## 3. Individual differences in category learning

In addition to sequence effect, whether there are individual differences concealed in the averaged data is our second concern. In the literature of category learning, heaps of individual differences are reported, providing us clues to understand individual participant's categorization strategy (Nosofsky et al., 1989; Johansen and Palmeri, 2002) and to evaluate models (Maddox and Ashby, 1993; Nosofsky et al., 1994). For instance, Yang and Lewandowsky (2004) examined human's category learning with multi-dimensional stimuli. Among all dimensions, one was the context dimension. In their experimental design, no matter the context dimension was attended to or not, participants could get perfect learning performance. The results showed a clear difference on categorization strategy. One group of participants learned to attended to the context dimension for categorization, whereas the other group did not. The modeling results further showed that ATRIUM (Erickson and Kruschke, 1998) (with rule plus exemplar) can account for the performance of both groups, whereas ALCOVE (Kruschke, 1992) (with exemplar only) had difficulty doing so. Thus, these authors suggested that multiple representations are used in categorization.

Perhaps, the most salient contribution of individual-difference analysis is to turn over our understanding of an old phenomenon. For instance, in order to examine the allocation of attention over stimulus dimensions during category learning, Lee and Wetzels (2010) reanalyzed the data of Kruschke (1993) study. In the condensation condition of this study, the category structure could be perfectly learned, if the information from two stimulus dimensions were integrated for categorization. Lee and Wetzels (2010) first fit GCM to the averaged data. The estimated attention weight on one dimension was about 0.55, suggesting that the participants did spread their attention equally on the two dimensions. However, when fitting GCM to the individual data, clear individual differences were observed. One group of participants focused their attention on one dimension, whereas the other group strongly attended to the other dimension. The averaged data disguised this fact and erroneously suggested that people evenly divided attention on the two dimensions when learning the condensed category structure. Therefore, the individual differences provide a more transparent understanding about how attention can be allocated during category learning.

Back to the issue of CVE. The past studies all reported the averaged data. As shown by Lee and Wetzels (2010)'s work, the averaged data might be not too much informative. Thus, it is reasonable to suspect that the non-CVE result reported in the past studies might actually contain the positive evidence of CVE as well. Thus, in this study, we would also like to examine the occurrence of CVE via the analysis of individual differences.

## 4. Experiments

According to previous discussions, we proposed two hypotheses to address the question why CVE was not consistently observed in category learning. First, there might be some individual differences buried under the averaged data. Perhaps those non-CVE reports actually included some participants who did show CVE and some others did not. Second, the classification for the mid-point item might be influenced by the preceding item, namely the sequence effect. As a result, the midpoint item may be classified as the high-variability category following some precedent and not following some others. In order to get rid of confounding from the regimen of experiment, we conducted this experiment in the conventional feedback-learning paradigm. All participants were asked to do the learning phase and then the transfer phase. The emphasis of data analysis was placed on verifying these two hypotheses.

**Figure 1:**
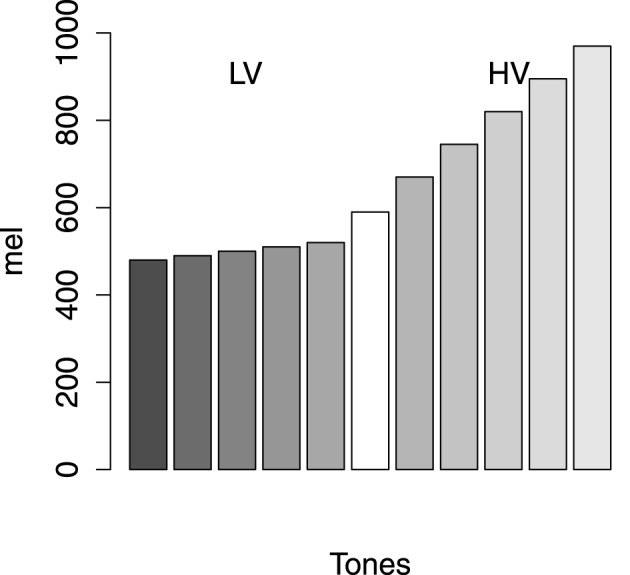
**The category structures**. HV, high variability; LV, low variability. Axis X represents tones and axis Y shows the *mel* of the stimuli.

In addition, we used single tones varying in frequency as stimuli in this experiment. In order to make the scale of stimuli equal in distance from one another, we transferred the frequency *f* to the psychological scale *mel*, $mel=1127log_{e}(\frac{f}{700}+1)$ (Steinberg, 1937; Stevens et al., 1937). The category structure was shown in Figure 1. There were five items in each category. The low-variability category (called Category 1) took the region between 480 and 520 *mel* and the high-variability category (called Category 2) took the region between 670 and 970 *mel*. The interval between the members of Category 1 was 10 *mel* and that of Category 2 was 75 *mel*. The critical item was the tone of 595 *mel*, which was denoted as the white bar in Figure 1. Therefore, if the probability of Category 1 for the critical item was less than 0.50, CVE occurred. All tones were played at a constant amplitude of 60 dB.

### 4.1. Methods

#### 4.1.1. Participants and apparatus

In total, 41 undergraduate students from National Chengchi University aged from 18 to 30 were recruited in this experiment. The whole experiment was conducted in a quiet dim booth. The display of stimuli, the procedure of testing, and the data collection were all controlled by the scripts of MATLAB on an IBM compatible PC. On average, every participant would finish this experiment in 30 min and got reimbursed with NTD$ 60 (≃ US$ 2) for their time and travel expense. Before doing the experiment, all participants were confirmed to be able to hear two extreme tones (i.e., 470 and 980 *mel*, covering the range of stimulus tones) each presented twice in a headset.

#### 4.1.2. Materials and procedure

Following the design of past studies (e.g., Sakamoto et al., 2008; Hsu and Griffiths, 2010), the two categories were defined as two uniform distributions. In the low-variability category, there were 5 tones equally spreading from 480 *mel* to 520 *meal* with 10 *mel* as the interval. In the high-variability category, there were also 5 tones equally spreading from 670 to 970 *mel* with 75 *mel* as the interval. There were 5 learning blocks, each of which was followed by a transfer block. Therefore, there were in total 10 blocks in this experiment. In the learning block, the 10 tones of the two categories were presented twice in random order. In the transfer block, the transfer stimuli consisted of 2 tones randomly sampled from each category and 1 critical item, which was 595 *mel* at the mid point between the edges of two categories. The transfer stimuli were presented once, except that the critical item was presented twice. Thus, there were 6 trials in total in the transfer block, which of course were presented in random order.

On each learning trial, a tone was presented to the participants from a headset for 1 s. After the stimulus disappeared, the participants were asked to predict which alien (i.e., Category 1 or Category 2) would make this sound by pressing the “s” key or the “;” key. Once the response was made, a “correct” or “wrong” feedback signal was presented on the computer screen for 500 ms. After 2 s, next trial began. The participants were instructed to do this task as accurately as they can. On each transfer trial, the procedure was the same as on the learning trial, except that there was no corrective feedback.

### 4.2. Results

#### 4.2.1. Learning phase

The participants learn the categories quite well. The accuracy in the first block is as high as 0.86 and it increases significantly to 0.95 in the fifth block, *F*_(4, 160)_ = 13.08, MSe = 0.003, *p* < 0.01. Clearly, this task is very easy to the participants.

#### 4.2.2. Transfer phase

**Figure 2:**
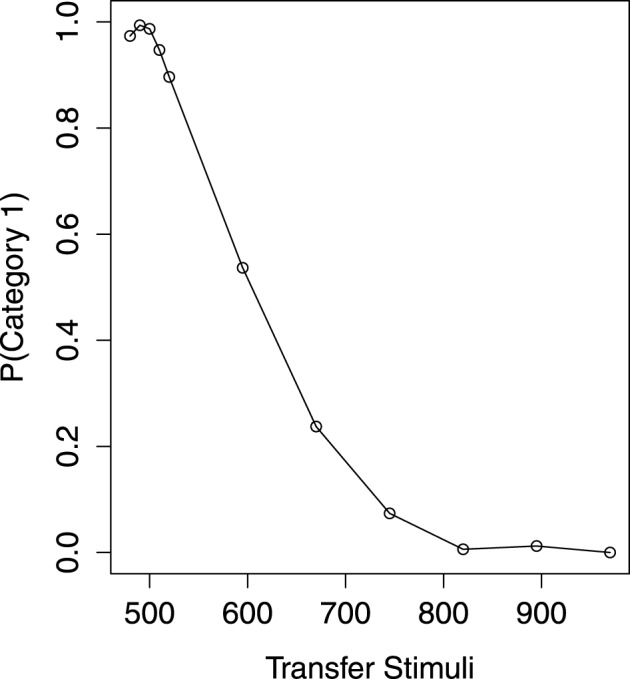
**The transfer performance**.

The mean probability of Category 1 on transfer item across five transfer blocks is shown in Figure 2. Axis X denotes the item *mel* and axis Y the probability of Category 1 predicted by the participants. For the items which have been presented in the learning phase, a Category (2) × Block (5) within-subject ANOVA shows that they are correctly classified as their own categories [*F*_(1, 40)_ = 3786, MSe = 0.02, *p* < 0.01]. However, the overall tendency to make a Category 1 response is influenced by the transfer block [*F*_(4, 160)_ = 6.564, MSe = 0.018, *p* < 0.01]. This is because there is a drop on the mean probability of Category 1 (0.46) in the final block. With no doubt, the response for the item from each category is not changed in different blocks [*F*_(4, 160)_ = 1.364, MSe = 0.016, *p* = 0.25]. Thus, the participants' categorization for the learning items is accurate and consistent through the transfer blocks.

Of most interest is how the participants would predict the category of the critical item. The mean probability of Category 1 on the critical item across five blocks is 0.54, which is not significantly different from 0.50 [*t*_(40)_ = 0.96, *p* = 0.34]. Thus, there is no evidence of CVE, as the critical item is not significantly classified as Category 2 (the high-variability category). However, the critical item is decreasingly classified as Category 1 in a linear trend from the first block [*p*_(Category1)_ = 0.68] to the fifth block [*p*_(Category1)_ = 0.41], with *F*_(1, 40)_ = 15.63, MSe = 0.15, *p* < 0.01. In the final block, the probability of Category 1 for the critical item is still not different from 0.50 [*t*_(40)_ = −1.42, *p* = 0.16].

#### 4.2.3. Sequence effect

As discussed in the previous section, how to classify an item might depend on which item it follows. In this experiment, the critical item is presented twice in every transfer block, once following a different item. Thus, it is reasonable to suspect that the critical item actually be classified as the high-variability category in one time, but as the low-variability category in another, so the aggregated result shows no CVE.

In order to verify this hypothesis, we examine for any sequence effect in our transfer data. Following the idea of the MAC strategy, we redefine the trials to four cases (C1+Up, C1+Down, C2+Up, and C2+Down)^1^, according to the category of the preceding item (Category 1 or Category 2) and the change of direction on frequency from the preceding item to the current one (Up or Down). One point is worth noting. In the transfer phase, there is no feedback, hence no correct answer for the preceding item. We substitute the participants' response for the category answer, due to the high learning accuracy they made in the experiment (mean = 0.94). If the participants rely on some long-term representations to do categorization (i.e., rule or exemplars of the two categories), the preceding category has nothing to do with the current response and so is the direction change between the frequencies of successive tones.

**Figure 3:**
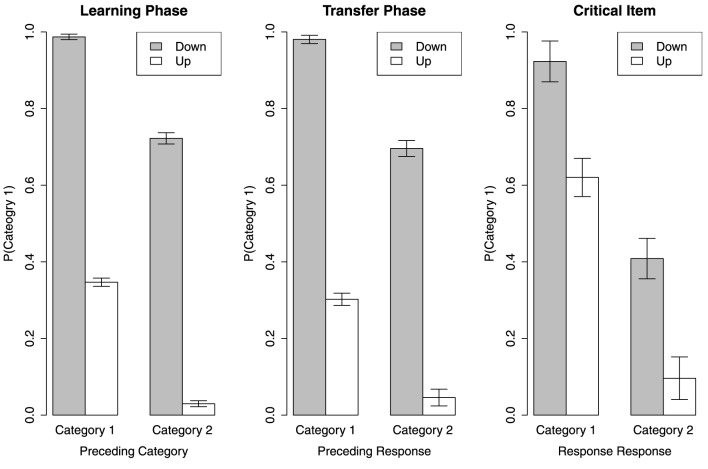
**Sequence effect in learning phase, transfer phase, and on the critical item**.

The results are revealed in Figure 3. See the left and middle panels for the overall results in the learning and transfer phases. Visual inspection shows when the direction of frequency change provides sufficient information, namely the cases of C1+Down and C2+Up, the participants strongly repeat the preceding category as the current response. For the cases of C1+Up and C2+Down, this tendency is not as strong. Across the learning and transfer phases, when the tone sounds higher than the preceding one, the participants tend to make a Category 2 response and when the tone sounds lower, they tend to make a Category 1 response [*F*_(1, 40)_ = 2695, MSe = 0.005, *p* < 0.01]. However, regardless of the direction of frequency change, the participants seem to repeat the preceding category as the current response to a certain extent that the main effect of the preceding category is significant [*F*_(1, 40)_ = 513.60, MSe = 0.01, *p* < 0.01]. The overall mean probability of Category 1 made for the current item is not different between the learning phase and the transfer phase [*F*_(1, 40)_ = 3.29, MSe = 0.006, *p* = 0.07]. The response pattern of the cases when the preceding item is from different categories is not different in different phases [*F*_(1, 40)_ = 1.03, MSe = 0.01, *p* = 0.32]. Also, the response pattern of the cases when the frequency change in different directions is not different in different phases [*F*_(1, 40)_ < 1]. There is no significant interaction effect between the preceding category and the direction of frequency change across all phases [*F*_(1, 40)_ < 1]. However, the three-way interaction effects between the experiment phase, the preceding category, and the frequency change direction is significant [*F*_(1, 40)_ = 5.55, MSe = 0.006, *p* < 0.05].

We also examine sequence effect on the critical item. See the right panel in Figure 3. Recall the critical item is actually higher in frequency than the members of Category 1 and lower than the members of Category 2. Thus, the cases of C1+Down and C2+Up theoretically do not exist. The two bars for these two cases represent the response made for the current critical item when the preceding item was also the critical item. However, for some participants who have never seen the critical item being presented twice in turn, we substitute the mean of the rest participants' data for the missing value. A Category (2) × Direction (2) within-subject ANOVA shows that the prediction for the critical item is influenced by the preceding response [*F*_(1, 40)_ = 139.2, MSe = 0.08, *p* < 0.1] and the change in direction of frequency from the preceding item [*F*_(1, 40)_ = 46.57, MSe = 0.08, *p* < 0.01]. However, there is no interaction effect between Category and Direction [*F*_(1, 40)_ < 1].

Although these results seem to be the evidence of sequence effect, the two cases C1+Down and C2+Up are actually not that informative. As the current item in these two cases can also be correctly categorized by a rule or by all exemplars of categories. Thus, the cases of C1+Up and C2+Down are our focus. It is clear that the participants tend to predict the current item as the category which is contrasting to the preceding one in the left and middle panels in Figure 3. However, this pattern is not held in the right panel for the critical item. In fact, the participants seem to predict the critical item as the same category of the preceding item, although the tendency is not strong. This is not surprising. When the critical item is in the C1+Up or C2+Down cases, similarity to the preceding item is the sole basis to predict its category. As the critical item is at the center position of all stimuli, the similarity between it and any other item is on average higher than that between any other pairs. Thus, in the C1+Up and C2+Down cases, the critical item would be more likely classified as the category of its preceding item. The analysis of sequence effect seems to suggest that the negative finding of CVE results from mixing all different influences brought by the preceding items in different testing situations. However, this conclusion is better not made quickly until we check out the individual differences.

### 4.3. Individual differences analysis

The sequence effect on the critical item may provide an explanation to why there is no CVE observed in the averaged data. However, we do not know whether this is a general case for all participants or there are some rule-use strategies^2^ mixed up in the averaged data. In fact, it is hard to detect those rule users by simply looking at the averaged sequence effect data. This is because their predictions for the critical item would be independent of the preceding item, that makes their influence as a constant added to the four categorization conditions. Therefore, we intend to investigate the individual differences by fitting the MAC model and the decision bound model^3^ to each participant's data. If the MAC model provides a better fit, the participant is regarded as a MAC strategy user. If the decision bound model provides a better fit, the participant is regarded as a rule user. Presumably, the participants who show CVE must be in the group of rule user. We can check out the probability of high-variability category predicted for the critical item to identify them. If there exist rule users, especially those who show CVE, the sequence effect should not be regarded as the reason for not observing CVE.

For each participant, we fit these two models to the transfer data separately. For the MAC model, only the specificity *c* is freely estimated. If the preceding item is from Category 2, the output will be transferred to *p*(1) = 1 − *p*(2) to make sure all MAC predictions are the probability of Category 1. For the decision bound model, the probability of Category 2 for item *X* is transferred from the area below the percept of *X* on the normal distribution with category boundary *b* as mean and perceptual error ϵ as standard deviation. The larger the covered area, the larger probability of Category 2 is^4^. The parameters *b* and ϵ are freely estimated. The stimulus values are normalized between 0 and 1 for modeling. The aim of parameter estimation is to maximize the likelihood of the model to predict the observed probability of Category 1 in the four categorization conditions (i.e., C1+Up, C1+Down, C2+Up, and C2+Down). The goodness of fit is *AIC* = −2*LogL+2N* (Aakike's Information Criterion; Akaike, 1974) with *N* = parameter number. The smaller *AIC* the better fit. The log likelihood is

(2) $$LogL=\sum_{i}log\left(\sum_{k}f_{ik}\right)!-\sum_{i}\sum_{k}\left(logf_{ik}\right)!+\sum_{i}\sum_{k}f_{ik}log\left(p_{ik}\right),$$

where *k* is the number of categories and *i* is the number of the categorization conditions.

**Figure 4:**
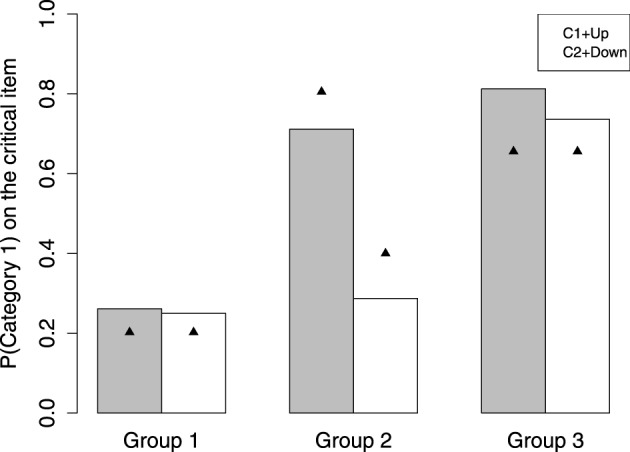
**The observed and predicted group difference on the critical item**.

According to the modeling results, the participants can be divided into three groups. See the *AIC* of each model for each group in Table 1. The group number is made in accordance with the tendency to predict the critical item as Category 1. Group 1 (*n* = 11) and Group 3 (*n* = 12) are consistent with the decision bound model, except that Group 1 tends not to classify the critical item as Category 1 and Group 3 strongly predicts the critical item as Category 1. Group 2 (*n* = 18) is identified as the MAC strategy user. The observed probability of Category 1 on the critical item made for each group is shown as the bars in Figure 4. Here we present the data collected in the C1+Up and C2+Down conditions. This is because the critical item is in between the two categories and it is always larger than a preceding item from Category 1 and smaller than a preceding item from Category 2. Thus, the cases of C1+Down and C2+Up are nearly impossible to happen for the critical item.

**Table 1:** **Model performance (AIC) on fit to transfer performance**.

	**MAC**	**Decision bound**
Group 1	52.34	43.50
Group 2	74.59	106.29
Group 3	61.84	53.34

Group 1 strongly classifies the critical item as Category 2, mean *p*(1) = 0.26, in either the C1+Up or C2+Down case. The performance of Group 1 in these two cases is not significantly different [*t*_(10)_ = 0.10, *p* = 0.92]. This result is better accommodated by the decision bound model. See the triangle in Figure 4, which represents the prediction of the winning model. For Group 1, the winning model is the decision bound model. See Table 2 for the parameter values, which provide best fits. The mean best-fit boundary *b* is 0.137, which equals 543 *mel*, locating in between the highest edge of Category 1 (520 *mel*) and the critical item (595 *mel*). Consequently, Group 1 shows CVE with no doubt.

**Table 2:** **Best-fitting parameter values**.

	**MAC**	**Decision bound**	
	***c***	***b***	**ϵ**
Group 1	1.32	0.137	0.035
Group 2	1.10	0.079	0.007
Group 3	1.06	0.244	0.029

Group 2 clearly shows sequence effect on classifying the critical item. On classifying the critical item, when following a Category 1 item [*p*(1) = 0.71 for C1+Up], Group 2 tends to make a response of Category 1, whereas when following a Category 2 item, Group 2 tends to make a response of Category 2 [*p*(1) = 0.28 for C2+Down]. The difference on probability of Category 1 between these two cases is significant [*t*_(17)_ = 3.89, *p* < 0.01]. The mean probability of Category 1 is about 0.50. The triangle shown for Group 2 in Figure 4 is the prediction of the MAC model.

Group 3 is a bit tricky, as these participants predict the critical item as Category 1 in the C1+Up case [*p*(1) = 0.81] and the C2+Down case [*p*(1) = 0.75]. For Group 3, the tendency to make classification for the critical item is not different in different categorization conditions [*t*_(11)_ = 0.91, *p* = 0.38]. The performance of Group 3 is better fit by the decision bound model. The mean best-fit boundary *b* is 0.244, which equals 599.56 *mel*. This boundary is larger than the critical item, hence predicting the critical item as Category 1. The decision bound model's prediction for Group 3 can be seen in Figure 4. However, this result presumably can also be accommodated by GCM. Since GCM would always predict the critical item as the low-variability category (i.e., Category 1), it is hard to say that Group 3 relies on rule or exemplars for categorization. One thing for sure is that Group 3 does not show CVE and does not rely on some short-term representation for categorization.

To sum up, a number of interesting findings in this experiment are listed as follow. First, CVE does occur in perceptual category learning (i.e., Group 1). Second, although some participants show CVE, some others do not, suggesting clear individual differences. Third, among those participants who do not show CVE, some take on the MAC strategy for categorization (i.e., Group 2) and some can be realized as doing categorization without considering the category variability (i.e., Group 3).

## 5. General discussion

In this study, we would like to figure out why CVE is seldom reported in the past studies. The analysis for the averaged data shows that there is no CVE. This is the same as what is reported in the past studies. We further examine two hypotheses for this result. One hypothesis is that the sequence effect in four categorization conditions, when being combined, would conceal CVE. The other is that the non-CVE report results from mixing up the uses of different categorization strategies, including the one which shows CVE. Although we find clear sequence effect, individual differences seem to provide a better account for why CVE is seldom reported. We fit the MAC model and the decision bound model to participants' transfer data with the attempt to detect any individual differences. The modeling results show three different groups. Group 1 shows CVE and is consistent with the decision bound model. Group 2 obviously adopts the MAC strategy, as supported by the clear sequence effect. Group 3 again is fit better by the decision bound model. However, this group tends to classify the critical item as the low-variability category.

In spite of positive evidence for CVE, a few constraints of this study need to mention. First, although it should be clear that Group 1 adopts rule for categorization and Group 2 adopts the MAC strategy, it is still not clear which representation, rule or exemplars, Group 3 forms for categorization. Second, we use only one item, namely the critical item, as the probe to examine CVE, that might decrease the power of our experiment. Instead of using one item, a line of novel items between two categories might be better as transfer items. Third, due to the randomization of trial orders, we cannot guarantee that the odds of each of the four categorization conditions (C1+Up, C1+Down, C2+Up, and C2+Down) are the same. Nonetheless, the implications of this study are discussed as follow.

### 5.1. Individual differences

Of our great interest is the individual differences revealed in this study. Group 1 classifies the critical item as the high-variability category, Group 2 classifies it as both categories depending on which item precedes it, and Group 3 classifies the critical item as the low-variability category. The reason why we have these individual differences might be relevant to the design of category structure and the individual participant's cognitive capacity. As to the category structure, the two categories in our experiment can be perfectly distinguished by a category boundary located on anywhere between them. When the boundary is put close to the low-variability category, we have Group 1, whereas when the boundary is put close to the high-variability category, we have Group 3.

Similarly, the study of Yang and Lewandowsky (2004) showed clear individual differences with a particular category structure, which could be represented by at least two different ways. The categories were constructed in a three-dimensional space, in which one dimension was context and could not directly predict the categories. The perfect learning performance could be achieved via either focusing on the related dimensions, ignoring the context dimension, to generate the true rule for categorization, or generating two different partial 2-D rules for categorization in different contexts. The participants did not know in advance this tricky part of the experiment, yet some of them learned to ignore context and some others learned to apply different rules for categorization in different contexts.

In a following study, the participants who relied on context to generate different rules for categorization were found to have a larger working memory capacity (operational span) than those who ignore context (Yang et al., 2006). This is reasonable, as attending more information does require more cognitive resource. In addition, a psychometric-approach study provides evidence that working memory capacity which is measured by the tasks of operational span, sentence span, memory updating, and spatial short-term memory is correlated with learning accuracy (*r* = 0.44) (Lewandowsky et al., 2012). Therefore, it is reasonable to expect that working memory capacity might have something to do with the individual differences we observed in this study. At least, we can expect that Group 2 might have a smaller working memory capacity than the other two groups. This is because they only need to retain the preceding item's information for current categorization, that consumes not too much cognitive capacity. The other two groups might need more efforts to generate the rule, which should be suitable for classifying all items. In the future study, the relationship between working memory capacity and category learning performance is worth investigating in more detail.

### 5.2. Short-term vs. long-term category representation

Most of contemporary models for category learning posit that categorization is accomplished by some long-term representation. For Group 1 and Group 3 in our study, it is true that some long-term representation must be formed for categorization. It could be a rule or exemplars of categories. Although Group 3 is fit better by the decision-bound model than the MAC model, it presumably is consistent with the prediction of GCM. Nonetheless, for Group 2, it is implied that the short-term exemplar memory might be relied on for categorization. Also, we should be able to find the evidence for the use of short-term representation in other experiments, as long as more one test trial is adopted. In fact, Navarro et al. (2013) recently ask the participants to learn the category structure, which varies along with learning trials. The task is not easy to learn, yet the participants' performance is above the chance level. They also report that the conventional exemplar model and prototype model cannot account for their data. Instead, their data can be fit by a heuristic model, which based on the preceding item to predict the category boundary for the next item. That is, the category boundary keeps shifting from one trail to the next. Together with their finding, the role of short-term representation in categorization should be more emphasized.

### 5.3. Conceptual vs. perceptual processing in categorization

Although the present study provides insights to why CVE was not reported in the perceptual categorization task, we do not think that these findings can properly benefit the conceptual categorization task, as the conceptual and perceptual processing differs substantially. In perceptual categorization, a rule can be defined mathematically as a boundary in the psychological space. Thus, as which category an item would be classified depends on which region in the psychological space the percept of this item locates in.

However, in conceptual categorization, a rule is often a logical statement such as “If necessary feature Y, then category X.” For example, an animal with a feature of “being born of cat parents” must be a cat, as our lay theory of animals demands that they must be of the same species as their parents. In the study of Rips (1989), the rule might state “If an object is more than 1 inch in diameter, it must be a PIZZA,” since quarters are severely restricted in size but pizzas are not. The feature “3-inches in diameter” is not characteristic of either PIZZA or QUARTER, but diagnostic of PIZZA, as a pizza can be as small as 3 inches in diameter. As shown in the study of Smith and Sloman (1994), when no characteristic features of QUARTER (e.g., silver colored) are present, the rule-based categorization is triggered and classifies the circular object with a 3-inches diameter as PIZZA. Clearly, CVE with conceptual categories is construed in a very different way.

In addition, in our study, the understanding of each category is established in the trial-by-trial learning experience, whereas the structure of conceptual category reflects our common knowledge of the world, which is acquired out of laboratory. Thus, the MAC strategy is not possible to be applied in the conceptual categorization task. On the other hand, it is expected that the sequence effect or the MAC strategy can be observed in other perceptual category learning tasks.

To sum up, our study provides evidence for the individual differences on classifying the critical item. This is regarded as one reason for why some studies report CVE but some others do not. Also, sequence effect is clearly observed in our experiment, which suggests the use of short-term representation for categorization. However, the success of the decision bound model suggests that long-term representation would also be used for categorization. Therefore, we find evidence for both short-term and long-term representation in a single study. However, it is still not clear why these individual differences occur, or how to induce a particular categorization strategy. These issues need to be addressed in future studies.

### Conflict of interest statement

The authors declare that the research was conducted in the absence of any commercial or financial relationships that could be construed as a potential conflict of interest.

## Footnotes

^1^Thus, the first trial is omitted.

^2^Since the exemplar model such as GCM is evident to have difficulties accounting for CVE, in order to detect the participants who actually show CVE, we search for them in rule users.

^3^GCM is precluded, as GCM is known unable to predict CVE.

^4^For fitting the participants data, probability of Category 2 would be transferred to probability of Category 1 by *p*(1) = 1 − *p*(2).
